# Phytochemicals and antioxidant capacity of some tropical edible plants

**DOI:** 10.5713/ajas.17.0903

**Published:** 2018-04-12

**Authors:** Heeok Hong, Jun-Hyeong Lee, Soo-Ki Kim

**Affiliations:** 1 Department of Medical Science, School of Medicine, Konkuk University, Seoul 05029, Korea; 2 Department of Animal Sciences and Technology, Konkuk University, Seoul 05029, Korea

**Keywords:** Phytochemical, Antioxidant Activity, Antibacterial Activity, Tropical Plants

## Abstract

**Objective:**

To find biological functions such as antibacterial and antioxidant activities in several tropical plants and to investigate the possibility of antibiotic substitute agents to prevent and treat diseases caused by pathogenic bacteria.

**Methods:**

Plants such as Poncirus trifoliata fruit (Makrut), Zingiber officinale Rosc (Khing), Areca catechu L. (Mak), Solanum melongena L. I (Makkhuayao), and Solanum melongena L. II (Makhurapro) were extracted by methanol, n-hexane, chloroform, ethyl acetate, butanol and water. The free radical scavenging activities were measured using 2-diphenyl-2-picryl hydrazyl photometric assay. Antibacterial activities with a minimum inhibitory concentration (MIC) were observed by agar diffusion assay against pathogenic strains of *Escherichia coli*, *Burkholderia* sp., *Haemopilus somnus*, *Haemopilus parasuis*, *Clostridium perfringens*, and *Pantoea agglomerans*.

**Results:**

Poncirus trifoliata fruit methanol extract showed antibacterial activities against gram-negative and gram-positive pathogens. Additionally, this showed the strongest antibacterial activity against *Burkholderia* sp. and *Haemopilus somnus* with MIC 131 μg/mL, respectively. Areca catechu L. water extract showed antibacterial activities against *Burkholderia* sp., *Haemopilus somnus*, and *Haemopilus parasuis*. The MIC value for *Haemopilus parasuis* was 105 μg/mL in this. Antioxidant activity of Zingiber officinale Rosc n-hexane extract showed 2.23 mg/mL effective concentration 50% (EC_50_) value was the highest activity among tropical plants extracts. Total polyphenol content in Zingiber officinale Rosc methanol extract was 48.4 μg/mL and flavonoid content was 22.1 μg/mL showed the highest values among tested plants extracts.

**Conclusion:**

Taken together, these results suggest that tropical plants used in this study may have a potential benefit as an alternative antibiotics agent through their antibacterial and antioxidant activities.

## INTRODUCTION

It is important to prevent and treat livestock diseases for improving productivity of livestock. Although antibiotics have played a key role for those, they have caused various side effects such as the generation of antibiotics-resistant bacteria [1]. As a result, consumers’ demand for eco-friendly agricultural products has been gradually increasing. Therefore, many countries are prohibiting the use of antibiotics.

Today, there has been an increasing concern to find alternative antibiotics substitutes in livestock diseases treatment particularly those of various natural products sources including plants to replace synthetic antibiotics used in livestock that are easy to obtain and have considerably few side effects [2,3]. Antibiotics substitutes derived from plants sources can promote the growth of livestock by preventing livestock diseases without causing tolerance to livestock and human, and they can reduce environmental pollution [4].

There are approximately 250,000 to 500,000 species of plants with pharmacological activity on this planet, but some plants have been investigated and reported for their antimicrobial activity and physiological activity [5–7]. Therefore, the screening of more plant and the research of antibacterial activity may play a critical role in the development of antibiotic substitute agents. Meanwhile, studies on plant extracts, such as subtropical and tropical plants have been actively conducted for the development of alternative agents [8,9]. Southeast Asian countries have an abundance of plant species because of their tropical climate that supports plants’ growth [10]. In our previous study, we observed the bioactivities of several tropical plants as follows. Citrus aurantifolia Swingle fruits and leaves showed the strongest antibacterial activity against *Haemopilus somnus* and *Burkholderia* sp., respectively. Piper sarmentosum Roxb leaves showed antibacterial activities against *Escherichia coli* (*E. coli*), *Burkholderia* sp. and *Haemopillus parsuis*. In addition, Citrus aurantifolia Swingle, Piper sarmentosum Roxb, Curcuma domestica Valeton roots, and Sesbania grandifloraL. have antioxidant effects [10].

Following the previous study, this experiment was conducted to screen other tropical plants such as *Poncirus trifoliata* fruit (Makrut), *Zingiber officinale* Rosc (Khing), *Areca catechu* L. (Mak), *Solanum melongena* L. I (Makkhuayao), and *Solanum melongena* L. II (Makhurapro).

*Poncirus trifoliata* fruit belonging to the Rutaceae family in the Citrus genus has been widely used in folk medicine as a remedy for inflammation, gastric and dysentery [11]. *Zingiber officinale* Rosc belonging to Zingiberaceae family is a medicinal plant widely used in treatment of bacterial infections because of their antimicrobial activity [12]. *Areca catechu* L. is one of the fruit plants of Palmaceae family and has been used to treat dysentery in East and North Asian countries [13]. Additionally, *Solanum melongena* L. cultivated worldwide throughout the tropics and subtropics belongs to the Leptostemonum Clade of the spiny solanums and is one of the most highly antioxidant vegetables, known as “eggplant” [14].

To date, there has been scant information on studies of alternative agents for the promotion of livestock health related to the bioactivity of these tropical plants.

Therefore, this study was conducted to investigate biological functions such as antibacterial and antioxidant activities and measure the content of polyphenol and flavonoid in other tropical plants to provide fundamental data for the antibiotics substitute agents that prevent and treat diseases caused by pathogenic bacteria.

## MATERIALS AND METHODS

### Preparation of plant extracts

*Poncirus trifoliata* fruit (Makrut), *Zingiber officinale* Rosc (Khing), *Areca catechu* L. (Mak), *Solanum melongena* L. I (Makkhuayao), and *Solanum melongena* L. II (Makhurapro) were selected for this study. They purchased from an authorized market (AOR TOR KOR) in Bangkok, Thailand. They were cut into smaller species and dried by using the dryer (Eyela WFO-600SD, Tokyo Rikakikai Co. Ltd, Tokyo, Japan) for three days and then ground finely with the grinder (DA-505, DAESUNG ARTLON, Paju, Korea). Those plants were extracted with water, methanol, n-hexane, chloroform, ethyl acetate, and butanol using the same method as in the previous study [10]. In briefly, a sample of 3 g was suspended in 27 mL methanol and was agitated with 150 rpm using the stirrer (KMC-130SH, Vision Scientific Co, Daejeon, Korea) for six hours, and then filtered and evaporated. The extract was resuspended with 2 mL methanol (methanol extract). Methanol extract 1 mL was mixed with n-hexane: H_2_O (1:1) and mixed vigorously and then separated by standing. Upper phase was collected (n-hexane extract) and same manner of previous step was processed to collect extracts of chloroform, ethyl acetate, and butanol in order.

### Antibacterial activity assay

Antibacterial activities of plant extracts were measured by the paper disk diffusion method, as described elsewhere [15]. Aliquots (25 μL) of each extract were dropped on sterilized paper with 6 mm of diameter and placed on suspended agar plate with pathogenic bacteria. And prepared plates were incubated at 37°C for 18 hours and appeared diameters of clear zone were measured. The pathogenic bacterial strains selected for this study were *E. coli*, *Burkholderia* sp., *Haemopillus somnus*, *Haemopillus parsuis*, *Clostridium perfringens*, and *Pantoea agglomerans*. They were obtained from the National Veterinary Research Quarantine Service in Korea.

### Determination of minimum inhibitory concentration

The minimum inhibitory concentration (MIC) values are used to determine susceptibilities of bacteria to drugs and evaluate the activity of new antibacterial agents [16]. According to the evaluation of antibacterial activities, MIC values of plant extracts were observed by broth micro-dilution method [17]. Plant extracts were diluted with sterilized Luria Bertani broth and dispensed in 96-well microtiter plates. Each tested strain was adjusted to 10^7^ to 10^8^ CFU/mL and diluted 1:100 ratio. And then they were added to the prepared each well and incubated at 37°C for 18 hours. The absorbance values were measured at 600 nm using microplate reader. The MIC value was determined as the lowest concentration of plant extracts showed no visible turbidity of the test strains.

### Antioxidant activity assay

The free radical scavenging activities of plant extracts were determined *in vitro* by 2-diphenyl-2-picryl hydrazyl (DPPH) photometric assay [18] with some modification. Briefly, 1 to 10 μL of each extract added to its solvent to make total volume of 100 μL and mixed with 900 μL of 0.04 mg/mL DPPH methanol solution and then stirred gently to react for 30 minutes at room temperature. The absorbance values were measured at 517 nm. Each solvent used in extraction was used as a negative control and *tert*-butyl hydroxytoluene was used as a positive control. The percentage antioxidant activity was calculated by comparing sample absorbance with negative controls and was plotted against sample concentrations. The antioxidant activities were expressed as EC_50_ value means the concentration at 50% of antioxidant activity.

### Total polyphenol contents of tropical plants

Total polyphenol contents of plant extracts were evaluated by the modified Folin-Ciocalteu method [19]. Na_2_CO_3_ solution (2 mL, 2%) was added to 0.1 mL extract and mixed for 10 minutes at room temperature. After the vortex, 0.1 mL of 50% Folin-Ciocalteu reagent was added and mixed again for 15 seconds, and then incubated for 30 minutes at 40°C to develop color. The absorbance was measured at 700 nm using UV-1601 spectrometer (Shimadzu, Kyoto, Japan). Plant extracts were evaluated at a final concentration of 1 mg/mL. Total polyphenol content of plant extracts was calculated as quercetin (25 to 500 ppm) using the following equation based on the calibration curve: y = 0.0033x–0.0111, R^2^ = 0.9979, where x was the absorbance and y was the quercetin equivalent (μg/mL) [20].

### Total flavonoid contents of tropical plants

Total flavonoids were estimated using the aluminum chloride colorimetric method [21]. Plant extract 0.5 mL was added to 1.5 mL of 95% ethanol. After mixture, 0.1 mL of 10% aluminum chloride, 0.1 mL of 1 M potassium acetate and 2.8 mL distilled water were added, respectively. They were mixed for 30 minutes at room temperature, the absorbance was measured at 415 nm using UV-1601 spectrometer (Shimazu, Japan). Extracts were evaluated at a final concentration of 1 mg/mL. Total flavonoid content of plant extracts was calculated as quercetin (5 to 100 ppm) using the following equation based on the calibration curve: y = 0.0068x–0.0165, R^2^ = 0.9974, where x was the absorbance and y was the quercetin equivalent (μg/mL).

## RESULTS AND DISCUSSION

### *In vitro* Antibacterial activity of tropical plants

As shown in Table 1, methanol extracts of *Poncirus trifoliata* fruits showed to have antibacterial activities against six animal pathogens such as *E. coli*, *Haemopillus somnus*, *Burkholderia* sp., *Haemopillus parsuis*, *Clostridium perfringens*, and *Pantoea agglomerans*. Especially, it showed the strongest antibacterial activity against *Haemopilus somnus*, a common disease-causing bacterium of boveine including respiratory disease, conjunctivitis, mastitis, and polyarthritis as a gram-negative pleomorphic bacterium worldwide [22,23]. Whereas other solvent extracts did not show antibacterial activities. *Zingiber officinale* Rosc, known as the medicinal plant [12], had moderate antibacterial activity (11.9 to 13.0 mm) against *Clostridium perfringens* in n-hexane, Chloroform, ethyl acetate, and butanol extract fractions. *Clostridium perfringens* as a gram-positive bacterium cause various forms of acute enteritis and fatal enterotoxemias in animals [24]. Ethyl acetate and butanol fractions of it appeared antibacterial activities (9.9 to 11.5 mm) against *Pantoea agglomerans* that may cause severe and occasionally fatal infections in animals and humans when introduced into the organs [25]. Water extract of *Areca catechu* L. had inhibitory effect on *Burkholderia* sp. (12.5 mm), *Haemopillus somnus* (12.3 mm), and *Haemopillus parsuis* (12.8 mm) and methanol of it showed antibacterial activity (12.5 mm) against *Burkholderia* sp. related to melioidosis in animals and humans [10]. It was reported that *Areca catechu* leaf extract had the inhibitory effects against inflammation *in vivo* and *vitro* [13]. According to Al-Bayati, *Areca catechu* nut water extract showed antibacterial activity against *E. coli* [26]. However, all solvent extracts of this showed no antibacterial activity against *E. coli* in this study.

*Solanum melongena* L. I (Makkhuayao) extract did not exhibit antibacterial activity against the tested pathogens in most solvent extracts but showed antibacterial activity (10.1 mm) against *Pantoea agglomerans* in the butanol fraction. And butanol extract of *Solanum melongena* L. II (Makhuapro) showed antibacterial activities against *Burkholderia* sp. (12.4 mm) and *Pantoea agglomerans* (9.7 mm), respectively.

### Minimum inhibitory concentration against animal pathogenic bacteria

MIC were tested against six animal pathogenic bacteria using broth micro-dilution method for plants extracts with antibacterial activities (Table 2). These results were like the antibacterial patterns shown by paper disc diffusion assay. Methanol extract of *Poncirus trifoliata* fruits appeared displayed MIC values with a range of 131 to 262 μg/mL against six pathogenic bacteria. Only its methanol extract showed MIC value 262 μg/mL and exhibited antibacterial activity against *E. coli*, a pathogen that infects cattle and swine colonies [27]. *Zingiber officinale* Rosc displayed the activity with MIC value of 240 μg/mL for the chloroform fraction and 690 μg/mL for n-hexane against *Clostridium perfringens*, a pathogen causing enterotoxenia of livestock [24]. Ethyl acetate extract of it showed MIC value of 247 μg/mL against *Haemopillus parsuis* and 1,980 μg/mL against *Pantoea agglomerans*. Butanol extracts of it had MIC values of 720 μg/mL, 1,140 μg/mL, and 1,140 μg/mL against *Burkholderia* sp., *Haemopillus parsuis*, and *Pantoea agglomerans*, respectively. *Areca catechu* L. had MIC values of 540 μg/mL for methanol, 1,680 μg/mL for water, and 240 μg/mL for butanol against *Burkholderia* sp. And butanol of it showed MIC value of 405 μg/mL against *Pantoea agglomerans*. Butanol extract of *Solanum melongena* L. I (Makkhuayao) showed MIC value of 397 μg/mL against *Pantoea agglomerans*, too. Butanol extract of *Solanum melongena* L. II (Makhuapro) displayed MIC values with 720 μg/mL against *Burkholderia* sp. and *Pantoea agglomerans*, respectively. As a result, MIC values of *Poncirus trifoliata* fruits methanol extract were 131 μg/mL for respiratory pathogens *Burkholderia* sp. and *Haemopillus somnus*, respectively and this plant exhibited antibacterial activity with the smallest amount among tested plants extracts. These results were like those of Rahman et al [28] that essential oil of this had inhibitory effects on plant pathogenic *Xanthomonas* spp with MIC values ranging 62.5 to 125 μg/mL.

### Total polyphenol content and flavonoid content

Total polyphenol content and flavonoid content were represented in Table 3. Total polyphenol amount of *Zingiber officinale* Rosc (Khing) methanol extract exhibited the highest vale as 48.4±0.00 μg/mL among all plant extracts, also the highest among them. This value was like that of Curcuma domestica Valeton (47.8 μg/mL) belonging to the same ginger family [10]. Conversely, Danciu et al [29] reported that total polyphenol content of this ethanol extract showed 16 mg GAE/g. Ghasemzadeh et al [30] recorded that polyphenol content in Malaysian *Zingiber officinale* root extract had 10.22 to 13.5 mg gallic acid/g. Thus, the polyphenol contents of *Zingiber officinale* in several studies represented various levels. The content of active phytochemicals is known to be highly influenced by extraction conditions such as solvent, temperature, and extraction time [31].

Most ginger plants probably contain a considerable number of polyphenols. Total flavonoid content of *Zingiber officinale* Rosc (Khing) methanol extract was 22.1±0.01 μg/mL, the largest among tested plants extracts. Those of the rest plants extracts showed 6.2 to 19.8 μg/mL and total flavonoid content in the methanol extracts of tested plants showed higher compared to those of other solvent extracts.

Those of methanol extracts in *Poncirus trifoliata* fruit and *Areca catechu* L. were 37.4±0.02 μg/mL and 38.7±0.01 μg/mL, respectively. Total polyphenol content of *Solanum melongena* L. I (Makkhuayao) and *Solanum melongena* L. II (Makhuapro) were 30.2±0.01 mg/mL and 34.8±0.01 mg/mL in butanol extracts, higher than those of other solvent extracts. In *Solanum melongena* L. II, the total polyphenol content of most solvent extracts were above 20 μg/mL and the total flavonoid contents were 10 μg/mL, and they showed to be slightly different among extracts except for Hexane extract (polyphenols 22.7 to 34.7 μg/mL vs flavonoids 10.9 to 15.9 μg/mL). *Poncirus trifoliata* fruit plant has been widely used in folk medicine as a remedy for inflammation, gastric and dysentery [11]. Additionally, several studies suggested that this had the ability of herbal medicine for anti-helicobacter pylori activity, anti-cancer, and anti-anaphylactic activities [11,32]. In this study, *Poncirus trifoliata* fruit in methanol extract contained 37.4 μg/mL of total polyphenol and 18.2 μg/mL of flavonoid, and thus showed antioxidant activity and above moderate anti-microbial effect with relative low MIC value. This extract may be beneficial to livestock health.

Although content of polyphenol and flavonoid in *Areca catechu* L. water extract were relatively lower compared those of other solvent extracts, antioxidant and antibacterial activities including MIC value of it were favorable. With these results, these bioactive components of it probably have different solubility depending on the extractive solvents used.

### Antioxidant activity of tropical plants

As shown Table 4, *Zingiber officinale* Rosc (Khing) exhibited antioxidant activities with the range of 1.10 to 2.23 mg/mL in n-hexane, chloroform, and butanol extracts.

*Zingiber officinale* Rosc, known as ginger, is the nutraceutical aromatic plant used as the spice and medicine worldwide [29]. This root extract has been used in Chinese and Indian traditional herbal medicine to treat indigestion, vomiting, arthritis, rheumatism, pains, cramps, fever, and infection [33]. It has been reported that this extract possesses valuable pharmacological phytocompounds attributed to antioxidant, antiinflammatory, immunomodulatory, antihyperglycemic, hypolipidemic, analgesic, and cardioprotective properties [33,34]. Tohma et al.’s results showed that ethanol extract of *Zingiber officinale* Rosc had better antioxidant activity than water extract of it. However, methanol and water extracts of this did not have antioxidant activities in this study [35]. Conversely, the antioxidant activity of n-hexane extract was the highest in this study, consistent with Račková ‘s results that n-hexane extract showed better DPPH radical-scavenging activity than other solvent extracts [36]. It was demonstrated that hydroethanolic extract of *Zingiber officinale* Rosc had strong free-radical reducing activity [33].

Antioxidant activity of *Poncirus trifoliata* fruit was 1.47±0.42 mg/mL in methanol extract but other extracts including water extract did not have antioxidant activities. Yu et al [37] reported that 70% ethanol extract of this showed more efficacy than water extract in the coronary from the pig. *Areca catechu* L grows throughout South, Southeast Asia and several Pacific Ocean islands [38]. This plant is the most commonly used as herbal medicine to treat dysentery and dysuria in Indonesia [13] and as an anthelmintic in humans and animals for a lengthy time in India and China [38]. *Areca catechu* L. showed antioxidant activity with 2.11±0.49 mg/mL in water extract with moderate inhibition (12.3 to 12.8 mm) on *Burkholderia* sp., *Haemopillus somnus*, and *Haemopillus parsuis*. Also, it was 1.06±0.11 mg/mL in methanol extract that appeared antibacterial activity against *Burkholderia* sp. These results were like Chavan’s results that in methanol and water extracts of it, total polyphenol content of *Areca catechu* L methanol extract showed higher than that of water extract and anti-oxidant activities showed relatively higher than those of other solvent extracts except acetone extract of it [39]. However, our result did not match the Hamsar et al.’s study result that *Areca catechu* methanol extract exhibited higher scavenging effect than water extract [38]. Plants have potential as antioxidants because they are rich sources of polyphenols including flavonoids. Although total polyphenol and flavonoids contents of methanol extract were higher than those of water extract, the EC_50_ value of water extract was higher than that of methanol extract in this study.

*Solanum melongena* L cultivated from tropical to temperate is one of the most consumed vegetables worldwide and one of the most highly antioxidant vegetables [14]. Antioxidant activities of *Solanum melongena* L. I (Makkhuayao) and *Solanum melongena* L. II (Makhuapro) were 1.85±0.27 mg/mL and 1.66±0.14 mg/mL in butanol extracts, respectively. Namely, it seems that high antioxidant activities are correlated with the higher content of polyphenol (30.2 and 34.8 μg/mL) and flavonoid (13.9 and 15.9 μg/mL) compared to those of other extracts. Polyphenols have been active antioxidants as plant secondary metabolites [29]. According several studies, the antioxidant activity of it was correlated with the abundance of polyphenols and flavonoids [40,41]. Specifically, there is a result that *Solanum melongena* peel extract has been significantly higher in free radical scavenging activity [14].

Taken together, the correlation between antibacterial and antioxidant activities and amounts of total polyphenol and flavonoid was not perfectly matched in this study. Nevertheless, methanol extract of *Poncirus trifoliata* fruit (Makrut) possessed antibacterial activities against all pathogens selected for this study. Especially, this extract possessed strong antibacterial activity against *Haemopillus somnus*. This extract also contained total polyphenol and flavonoid higher amounts than the other solvents extracts and showed the antioxidant activity in proportion to these contents. In addition, hexane, chloroform, and butanol extracs of *Zingiber officinale* Rosc (Khing), water extract of *Areca catechu* L. (Mak), and butanol extract of *Solanum melongena* L. II (Makhuapro) showed moderate antibacterial and antioxidant activities.

The objective of this study was to identify suitable antibiotics using several tropical plants to improve productivity owing to disease prevention and treatment in livestock. Based on these results in this study, tropical plants have contained abundantly antioxidants such as polyphenol and flavonoids and biological functions such as antimicrobial and antioxidant activities have been effective. Tropical plants extract used in this study are possible substitute antibiotics. Therefore, further research on more tropical plants are needed to screen for their bioactivities and enable development of better antibiotic substitutes for animal health. The probable reason for this could be the fact that they may be used as a resource to improve livestock health and can be easily used in such circumstances where they are widely available and can be harvested in large quantities throughout the year.

## Figures and Tables

**Table 1:** Antibacterial activities of tropical plants extract against animal pathogenic bacteria

Plant tested	Pathogenic bacteria	Solvent fraction1)					

		A	B	C	D	E	F

		Clear zone2) (mm)					
*Poncirus trifoliate* fruit (Makrut)	*Escherichia coli*	-3)	10.5±0.38	-	-	-	-
	*Burkholderia* sp.	-	12.8±0.29	-	-	-	-
	*Haemopillus somnus*	-	16.8±0.09	-	-	-	-
	*Haemopillus parsuis*	-	11.8±0.21	-	-	-	-
	*Clostridium perfringens*	-	12.4±0.26	-	-	-	-
	*Pantoea agglomerans*	-	11.7±0.32	-	-	-	-
*Zingiber officinale* Rosc (Khing)	*Escherichia coli*	-	-	-	-	-	-
	*Burkholderia* sp.	-	-	-	-	-	-
	*Haemopillus somnus*	-	-	-	-	-	-
	*Haemopillus parsuis*	-	-	-	-	-	-
	*Clostridium perfringens*	-	-	13.0±0.19	12.6±0.33	12.1±0.27	11.9±0.06
	*Pantoea agglomerans*	-	-	-	-	11.5±0.07	9.9±0.34
*Areca catechu* L. (Mak)	*Escherichia coli*	-	-	-	-	-	-
	*Burkholderia* sp.	12.5±0.18	12.5±0.12	-	-	-	+
	*Haemopillus somnus*	12.3±0.31	-	-	-	-	-
	*Haemopillus parsuis*	12.8±0.15	-	-	-	-	-
	*Clostridium perfringens*	-	-	-	-	-	-
	*Pantoea agglomerans*	-	-	-	-	-	10.3±0.29
*Solanum melongena* L. I (Makkhuayao)	*Escherichia coli*	-	-	-	-	-	-
	*Burkholderia* sp.	-	-	-	-	-	-
	*Haemopillus somnus*	-	-	-	-	-	-
	*Haemopillus parsuis*	-	-	-	-	-	-
	*Clostridium perfringens*	-	-	-	-	-	-
	*Pantoea agglomerans*	-	-	-	-	-	10.1±0.15
*Solanum melongena* L. II (Makhuapro)	*Escherichia coli*	-	-	-	-	-	-
	*Burkholderia* sp.	-	-	-	-	-	12.4±0.30
	*Haemopillus somnus*	-	-	-	-	-	-
	*Haemopillus parsuis*	-	-	-	-	-	-
	*Clostridium perfringens*	-	-	-	-	-	-
	*Pantoea agglomerans*	-	-	-	-	-	9.7±0.06

1) Solvent fractions: A, water; B, methanol; C, n-hexane; D, chloroform; E, ethylacetate; F, butanol.

2) Mean±standard error (n = 3).

3) Antibacterial activities: -, no inhibition (8 mm); very slight inhibition (9 to 11 mm); moderate inhibition (11 to 13 mm); strong inhibition (13 to 17 mm).

**Table 2:** Minimum inhibitory concentration of tropical plants extracts against various microorganisms

Plant tested	Solvent fraction	Test pathogenic bacterial strains for antibacterial activities					

		E. coli	Burkholderia sp.	Haemopillus somnus	Haemopillus parasuis	Clostridium perfringens	Pantoea agglomerans

		Minimum inhibitory concentration (μg/mL)					
*Poncirus trifoliata* fruit (Makrut)	Methanol	262	131	131	262	262	262
*Zingiber officinale* Rosc (Khing)	*n*-Hexane	ND	ND	ND	ND	690	ND
	Chloroform	ND	ND	ND	ND	240	ND
	Ethylacetate	ND	ND	ND	247	ND	1,980
	Buthanol	ND	720	ND	1,140	ND	1,140
*Areca catechu* L. (Mak)	Methanol	ND	540	ND	ND	ND	ND
	Water	ND	1,680	210	105	ND	ND
	Buthanol	ND	240	ND	ND	ND	405
*Solanum melongena* L. (Makkhuayao)	Buthanol	ND	ND	ND	ND	ND	397
*Solanum melongena* L. (Makhuapro)	Buthanol	ND	720	ND	ND	ND	720

ND, not determined.

**Table 3:** Total polyphenol and flavonoid contents from tropical plant extracts

Plant tested	Part used	Solvent fraction	Contents (μg/mL)1)	

			Total polyphenol	Total flavonoid
*Poncirus trifoliata* fruit (Makrut)	Fruit	Methanol	37.4±0.0233	18.2±0.0021
		*n*-Hexane	23.9±0.0071	11.7±0.0071
		Chloroform	21.4±0.0092	9.7±0.0064
		Ethylacetate	26.8±0.0049	12.8±0.0049
		Buthanol	16.8±0.0064	8.9±0.0071
		Water	15.8±0.0028	11.8±0.0064
*Zingiber officinale* Rosc (Khing)	Rhizome	Methanol	48.4±0.0042	22.1±0.0106
		*n*-Hexane	25.8±0.0057	11.7±0.0156
		Chloroform	21.7±0.0035	9.9±0.0028
		Ethylacetate	13.0±0.0042	6.2±0.0049
		Buthanol	14.5±0.0028	7.6±0.0057
		Water	17.3±0.0042	8.8±0.0064
*Areca catechu* L. (Mak)	Fruit	Methanol	38.7±0.0078	19.8±0.0042
		*n*-Hexane	14.5±0.0028	7.9±0.0070
		Chloroform	23.9±0.0071	10.7±0.0078
		Ethylacetate	28.9±0.0049	13.7±0.0064
		Buthanol	13.9±0.0057	7.6±0.0042
		Water	20.3±0.0071	9.3±0.0078
*Solanum melongena* L. (Makkhuayao)	Fruit	Methanol	26.4±0.0045	14.3±0.0028
		*n*-Hexane	23.6±0.0057	10.7±0.0042
		Chloroform	19.1±0.0156	9.5±0.0057
		Ethylacetate	22.9±0.0120	10.9±0.0064
		Buthanol	30.2±0.0064	13.9±0.0085
		Water	17.8±0.0057	7.9±0.0057
*Solanum melongena* L. (Makhuapro)	Fruit	Methanol	28.8±0.0028	13.4±0.0092
		*n*-Hexane	19.4±0.0085	9.3±0.0064
		Chloroform	23.0±0.0014	12.6±0.0014
		Ethylacetate	22.7±0.0028	10.9±0.0021
		Buthanol	34.8±0.0184	15.9±0.0091
		Water	24.1±0.0035	11.8±0.0071

1) Mean±standard error (n = 3).

**Table 4:** Antioxidant activities of tropical plant extracts

Plant tested	Free radical scavenging activity1)	

	Solvent fraction	EC_50_ value (mg/mL)
*Poncirus trifoliata* fruit (Makrut)	Methanol	1.47±0.4222)
*Zingiber officinale* Rosc (Khing)	*n*-Hexane	2.23±1.48
	Chloroform	1.23±0.39
	Ethylacetate	ND
	Buthanol	1.10±0.40
*Areca catechu* L. (Mak)	Methanol	1.06±0.11
	Water	2.11±0.49
	Buthanol	ND
*Solanum melongena* L. (Makkhuayao)	Buthanol	1.85±0.27
*Solanum melongena* L. (Makhuapro)	Buthanol	1.66±0.14

ND, not detected; DPPH, 2-diphenyl-2-picryl hydrazyl; BHT, *tert*-butyl hydroxytoluene.

1) Free radical scavenging activity was determined by DPPH photometric assay and described in EC_50_ value which means the concentration at 50% of antioxidant activity. BHT was used as a positive control showy EC_50_ of 0.3±0.04 mg/mL.

2) Mean±standard error (n = 3).

## References

[1] Abimbola KA, Obi CL, Alabi SA, Olukoya DK, Ndip RN (1993). Current status on biotyping antibiogram and plasmid profiles of *E. coli* isolates. East Afr Med J.

[2] Khulbe K, Sati SC (2009). Antibacterial activity of *Boenninghausenia albiflora* Reichb. (Rutaceae). Afr J Biotechnol.

[3] Islam K, Rowsni AA, Khan M, Kabir S (2014). Antimicrobial activity of ginger (*Zingiber officinale*) extracts against food-borne pathogenic bacteria. Int J Sci Environ Technol.

[4] Wegener HC (2003). Antibiotics in animal feed and their role in resistance development. Curr Opin Microbiol.

[5] Cowan MM (1999). Plant products as antimicrobial agents. Clin Microbiol Rev.

[6] Gautam R, Saklani A, Jachak SM (2007). Indian medicinal plants as a source of antimycobacterial agents. J Ethnopharmacol.

[7] Prasad SK, Laloo D, Kumar M, Hemalatha S (2013). Antidiarrhoeal evaluation of root extract, its bioactive fraction, and lupinifolin isolated from *Eriosema chinense*. Planta Med.

[8] Esquenazi D, Wigg MD, Miranda MM (2002). Antimicrobial and antiviral activities of polyphenolics from *Cocos nucifera* Linn. (Palmae) husk fiber extract. Res Microbiol.

[9] Wannissorn B, Jarikasem S, Siriwangchai T, Thubthimthed S (2005). Antibacterial properties of essential oils from Thai medicinal plants. Fitoterapia.

[10] Lee JH, Cho S, Paik HD (2014). Investigation on antibacterial and antioxidant activities, phenolic and flavonoid contents of some Thai edible plants as an alternative for antibiotics. Asian-Australas J Anim Sci.

[11] Yi JM, Kim MS, Koo HN (2004). *Poncirus trifoliata* fruit induces apoptosis in human promyelocytic leukemia cells. Clin Chim Acta.

[12] Chen IN, Chang CC, Ng CC (2008). Antioxidant and antimicrobial activity of Zingiberaceous plants in Taiwan. Plants Foods Hum Nutr.

[13] Lee KP, Sudjarwo GW, Kim JS (2014). The anti-inflammatory effect of Indonesian *Areca catechu* leaf extract *in vitro* and *in vivo*. Nutr Res Pract.

[14] Bernardo JS, Sagum RS (2016). Eggplant (*Solanum melongena* L.) peel as a potential functional ingredient in pan de sal. J Nutr Food Sci.

[15] Hong H, Niu KM, Lee JH (2016). Characteristics of Chinese chives (*Allium tuberosum*) fermented by *Leuconostoc mesenteroides*. Appl Biol Chem.

[16] Wiegand I, Hilpert K, Hancock RE (2008). Agar and broth dilution methods to determine the minimal inhibitory concentration (MIC) of antimicrobial substances. Nat Protoc.

[17] Alviano WS, Alviano DS, Diniz CG (2008). *In vitro* antioxidant potential of medicinal plant extracts and their activities against oral bacteria based on Brazilian folk medicine. Arch Oral Biol.

[18] Tadić VM, Dobrić S, Marković GM (2008). Anti-inflammatory, gastroprotective, free-radical-scavenging, and antimicrobial activities of hawthorn berries ethanol extract. J Agric Food Chem.

[19] Wolfe K, Wu X, Liu RH (2003). Antioxidant activity of apple peels. J Agric Food Chem.

[20] Kiselova Y, Ivanova D, Chervenkov T (2006). Correlation between the *in vitro* antioxidant activity and polyphenol content of aqueous extracts from Bulgarian herbs. Phytother Res.

[21] Ordon AAL, Gomez JD, Vattuone MA, Isla MI (2006). Antioxidant activities of *Sechium edule* (Jacq.) Swart extracts. Food Chem.

[22] Harris FW, Janzen ED (1989). The *Haemophilus somnus* disease complex (Hemophilosis): a review. Can Vet J.

[23] Sandal I, Inzana TJ (2010). A genomic window into the virulence of *Histophilus somni*. Trends Microbiol.

[24] Niilo L (1980). *Clostridium perfringens* in animal disease: a review of current knowledge. Can Vet J.

[25] Cruz AT, Cazacu AC, Allen CH (2007). *Pantoea agglomerans*, a plant pathogen causing human disease. J Clin Microbiol.

[26] Al-Bayati NJM (2016). *In*-*vitro* antibacterial and antifungal effect of areca nut extract. Res J Pharm Biol Chem Sci.

[27] Li R, He L, Hao L (2013). Genotypic and phenotypic characterization of antimicrobial-resistant *Escherichia coli* from farm-raised diarrheic sika deer in Northeastern China. Plos One.

[28] Rahman A, Islam R, Al-Reza SM, Kang SC (2014). *In vitro* control of plant pathogenic *Xanthomonas* spp. Using *Poncirus trifoliata* rafin. EXCLI J.

[29] Danciu C, Vlaia L, Fetea F (2015). Evaluation of phenolic profile, antioxidant and anticancer potential of two main representants of Zingiberaceae family against B164A5 murine melanoma cells. Biol Res.

[30] Ghasemzadeh A, Jaafar HZE, Rahmat A (2010). Antioxidant activities, total phenolics and flavonoids content in two varieties of Malaysia young ginger (*Zingiber officinale* Roscoe). Molecules.

[31] Sasidharan S, Chen Y, Saravanan D, Sundram KM, Yoga Latha L (2011). Extraction, isolation and characterization of bioactive compounds from plants’ extracts. Afr J Tradit Complement Altern Med.

[32] Kim DH, Bae EA, Han MJ (1999). Anti-Helicobacter pylori activity of the metabolites of poncirin from *Poncirus trifoliata* by human intestinal bacteria. Biol Pharm Bull.

[33] Ali BH, Blunden G, Tanira MO, Nemmar A (2008). Some phytochemical, pharmacological and toxicological properties of ginger (*Zingiber officinale* Roscoe): a review of recent research. Food Chem Toxicol.

[34] Siddaraju MN, Dharmesh SM (2007). Inhibition of gastric H+,K+ -ATPase and *Helicobacter pylori* growth by phenolic antioxidants of *Zingiber officinale*. Mol Nutr Food Res.

[35] Tohma H, Gülçin L, Bursal E (2017). Antioxidant activity and phenolic compounds of ginger (*Zingiber officinale* Rosc.) determined by HPLC-MS/MS. J Food Meas Charact.

[36] Račková L, Cupáková M, Ťažký A (2013). Redox properties of ginger extracts: Perspectives of use of *Zingiber officinale* Rosc. as antidiabetic agent. Interdiscip Toxicol.

[37] Yu DJ, Jun JH, Kim TJ (2015). The relaxing effect of *Poncirus fructus* and its flavonoid content on porcine coronary artery. Lab Anim Res.

[38] Hamsar MN, Ismail S, Mordi MN, Ramanathan S, Mansor SM (2011). Antioxidant activity and the effect of different parts of *areca catechu* extracts on Glutathione-S-Transferase activity *in vitro*. Free Radic Antioxid.

[39] Chavan YV, Singhal RS (2013). Separation of polyphenols and arecoline from areca nut (*Areca catechu* L.) by solvent extraction, its antioxidant activity, and identification of polyphenols. J Sci Food Agric.

[40] Akanitapicha P, Phraibung K, Nuchklang K, Prompitakkul S (2010). Antioxidant and hepatoprotective activity of five eggplant varieties. Food Chem Toxicol.

[41] Azuma K, Ohyama A, Ippoushi K (2008). Structures and antioxidant activity of anthocyanins in many accessions of eggplant and its related species. J Agric Food Chem.

